# High-resolution calcareous nannoplankton palaeoecology as a proxy for small-scale environmental changes in the Early Miocene

**DOI:** 10.1016/j.marmicro.2014.06.005

**Published:** 2014-09

**Authors:** Gerald Auer, Werner E. Piller, Mathias Harzhauser

**Affiliations:** a Institute for Earth Sciences, University of Graz, NAWI Graz, Heinrichstrasse 26, 8010 Graz, Austria; b Natural History Museum Vienna, Geological–Paleontological Department, Burgring 7, 1010 Vienna, Austria

**Keywords:** High-resolution, Palaeoecology, Calcareous nannoplankton, Small-scale environmental change, Early Miocene, North Alpine Foreland Basin

## Abstract

Within a 5.5-m-thick succession of upper Burdigalian (CNP-zone NN4) shallow neritic sediments from the North Alpine Foreland Basin in Lower Austria a high-resolution section of finely laminated sediment with a thickness of 940.5 mm was logged. The section was continuously sampled, resulting in 100 samples, covering a thickness of ~ 10 mm each. An integrated approach was applied to these samples in order to study proxy records including calcareous nannoplankton, geochemical and geophysical data.

Multivariate statistics on the autochthonous assemblage were used to evaluate the ecological preferences of each taxon and to rule out possible contamination of the signal by taphonomic processes. In order to assess changes in the assemblage composition throughout the section, three taphogroups were defined using both the autochthonous and allochthonous nannofossils. Based on the distribution of these taphogroups five distinct intervals were defined that are indicative of centennial to decadal changes in palaeoenvironmental conditions. Combining these results with other proxies (geochemistry, geophysics) we were able to reconstruct short-term, small-scale variations in terms of temperature, primary productivity, bottom water oxygenation, organic matter flux, freshwater influx and changes in relative sea level in a highly dynamic shallow marine setting.

This study represents the first such high-resolution analysis performed on a marine succession of late Burdigalian age. It is also a first attempt to analyse outcrop data on such a high-resolution, sub-Milankovitch scale, with respect to calcareous nannoplankton in conjunction with geochemical and sedimentological data.

## Highlights

• Decadal scale environmental changes in an Early Miocene upwelling zone.
• First ultra-high-resolution study on Miocene calcareous nannoplankton.
• Nannofossils as a proxy for late Burdigalian small-scale environmental changes.
• Description of a Paratethyan shelf setting of the North Alpine Foreland Basin.
• Integrated approach using palaeoecological and geochemical proxy record.

## 1 Introduction

Although geosciences are a major player in the climate change debate, high-resolution palaeoecological and palaeoenvironmental studies on a decadal to millennial scale are mostly restricted to geologically young sediments, in particular, to those of the Quaternary (e.g., Meyers and Arnaboldi, 2005, Kloosterboer-van Hoeve et al., 2006, Mudie et al., 2007, Sprovieri et al., 2012). Undisturbed and laminated sediments without bioturbation are best suited for this type of study. Examples from the pre-Pleistocene originate mostly from palaeolakes where laminated or varved sediments are relatively abundant (Lindqvist and Lee, 2009, Lenz et al., 2010, Gross et al., 2011, Kern et al., 2012, Kern et al., 2013). In pre-Pleistocene marine sediments palaeoecological studies based on palaeobiological proxies have never been performed on a comparable resolution. Principally, only a few proxies are applicable in high-resolution studies. In addition to geochemical and geophysical proxies only microfossils can be used for this type of study because of their small size. Calcareous nannofossils fulfil these requirements best, however, they are mostly well known for their excellent stratigraphic potential in the Mesozoic and Cenozoic, which is expressed in standard biostratigraphic zonations (e.g., Bown, 1998 and references therein). Although a deep understanding of their ecology is still lacking, general inferences on the ecological preferences of many taxa can still be drawn from a careful study of nannoplankton literature data (e.g., Ziveri et al., 2004). Combined with statistical methods, especially cluster analysis, these data show promise as a powerful tool for ecological interpretations of nannofossil assemblages (e.g., Spezzaferri and Ćorić, 2001, Couapel et al., 2007), even with the severe limitations imposed on the current knowledge of nannoplankton ecology.

In particular, for high-resolution studies calcareous nannoplankton have only been used in Pleistocene to Holocene deposits (Negri and Giunta, 2001, Álvarez et al., 2005, Mertens et al., 2009). In Miocene surface outcrops it has never been studied with a decadal to millennial resolution.

In order to demonstrate that calcareous nannoplankton assemblages are an excellent proxy for high-resolution palaeoenvironmental reconstructions we use laminated marine sediments of late Burdigalian age from the Central Paratethys with high sedimentation rates (estimated as 500 mm kyr^− 1^ based on calculations in similar settings; Hohenegger and Wagreich, 2011). Together with the distribution of nannoplankton assemblages we also analyse geochemical and sedimentological proxies. The results show that Early Miocene coccolithophores were highly sensitive to changes in environmental conditions and thus allow ecological reconstructions with a high temporal resolution.

## 2 Geological setting

**Fig. 1:**
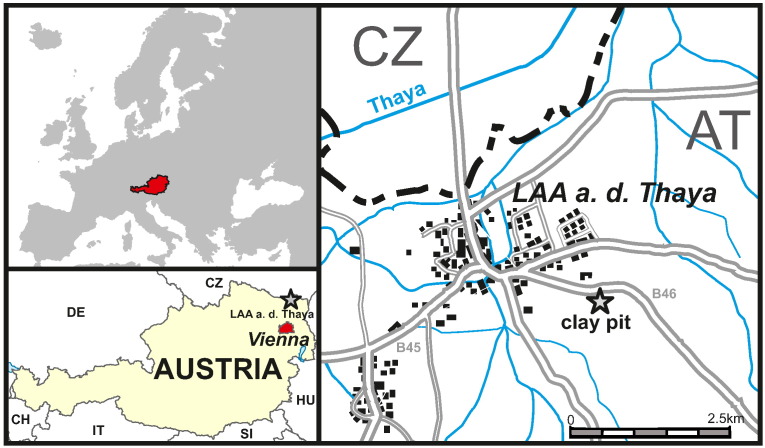
Location map showing the geographical position of the clay pit near Laa an der Thaya in Lower Austria where the studied section is located.

**Fig. 2:**
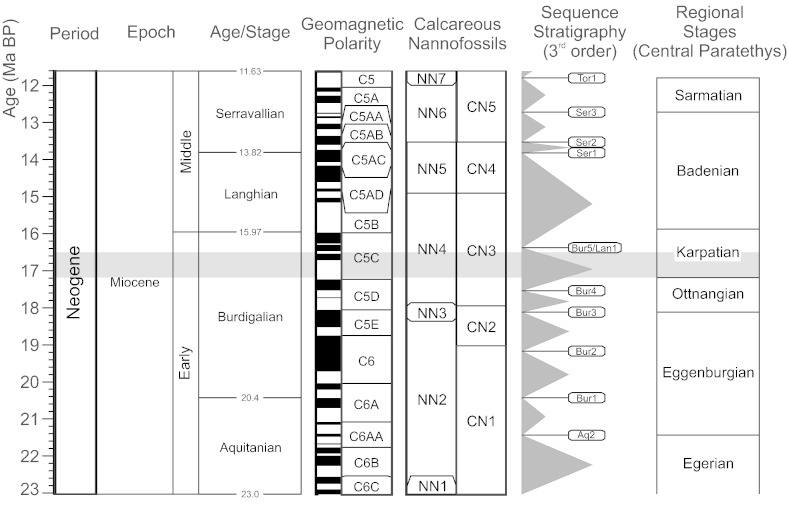
Global and regional Miocene chronostratigraphy and nannoplankton stratigraphy. Global chronostratigraphy follows Gradstein et al. (2012 and references therein). Sequence stratigraphy after Hardenbol et al. (1988). Regional stratigraphy of the Central Paratethys after Harzhauser and Piller (2007) with calibrated ages of the Ottnangian and Karpatian after Grunert et al. (2010b) and Dellmour and Harzhauser (2012).

The studied section is located in an abandoned clay pit ESE of Laa an der Thaya in Lower Austria (48° 43′ 0.84″N, 16° 24′ 45.18″ E), about 50 km north of Vienna (Fig. 1). Palaeogeographically, the area of deposition was located within the North Alpine Foreland Basin (NAFB), which was part of the Central Paratethys during the Oligocene and Miocene (Harzhauser and Piller, 2007, Piller et al., 2007). In the studied clay pit sediments of the Laa Formation are exposed. This formation is an upper Burdigalian lithostratigraphic unit, with a thickness of up to 1000 m (as recorded in well logs near the outcrop), which displays a general coarsening upward trend and corresponds to the lower and middle Karpatian of the regional chronostratigraphic scheme of the Central Paratethys (Piller et al., 2007; Fig. 2). The total geochronologic range of the Karpatian is 17.2–15.9 Ma (Grunert et al., 2010b, Grunert et al., 2012; Fig. 2). The Laa Formation unconformably overlies Ottnangian sediments (Nehyba and Petrová, 2000, Roetzel and Schnabel, 2002, Adamek et al., 2003). The marine sediments of the Laa Formation are composed of calcareous, laminated, greenish to brownish grey, micaceous, silty shales with thin fine sand intercalations (Roetzel and Schnabel, 2002). Dellmour and Harzhauser (2012) suggest an age of c. 17.2–16.5 Ma for the Laa Formation. Due to the isolated occurrence of the studied outcrop no precise position of these sediments within the formation can be given.

Benthic foraminiferal assemblages and the P/B-ratio indicate water depths between 100 and 200 m (inner to outer shelf environment) (Spezzaferri and Ćorić, 2001). Sandy intercalations are interpreted as episodic storm events (Roetzel and Schnabel, 2002). Nannoplankton assemblages indicate cool to temperate, nutrient-rich surface-water conditions that triggered the formation of dysoxic bottom waters (Spezzaferri and Ćorić, 2001, Spezzaferri et al., 2002). Upwelling conditions have been reported for the study area as well as for other parts of the North Alpine Foreland Basin (Spezzaferri and Ćorić, 2001, Roetzel et al., 2007).

The entire Laa Formation is dated to Neogene Nannoplankton Zone 4 (NN4) (Martini, 1971) or Mediterranean Neogene Nannoplankton Zone 4a (MNN4a; Fornaciari and Rio, 1996, Fornaciari et al., 1996) (Spezzaferri and Ćorić, 2001, Spezzaferri et al., 2002, Adamek et al., 2003, Svabenicka et al., 2003). Both of which correspond to Calcareous Nannofossil Miocene biozone 6 (CNM6) of Backman et al. (2012). The calcareous nannofossil assemblages are of low to moderate diversity and contain a very high amount (up to 45%) of reworked (Palaeogene and Cretaceous) specimens (Spezzaferri and Ćorić, 2001).

## 3 Material and methods

### 3.1 Sampling and field methods

**Fig. 3:**
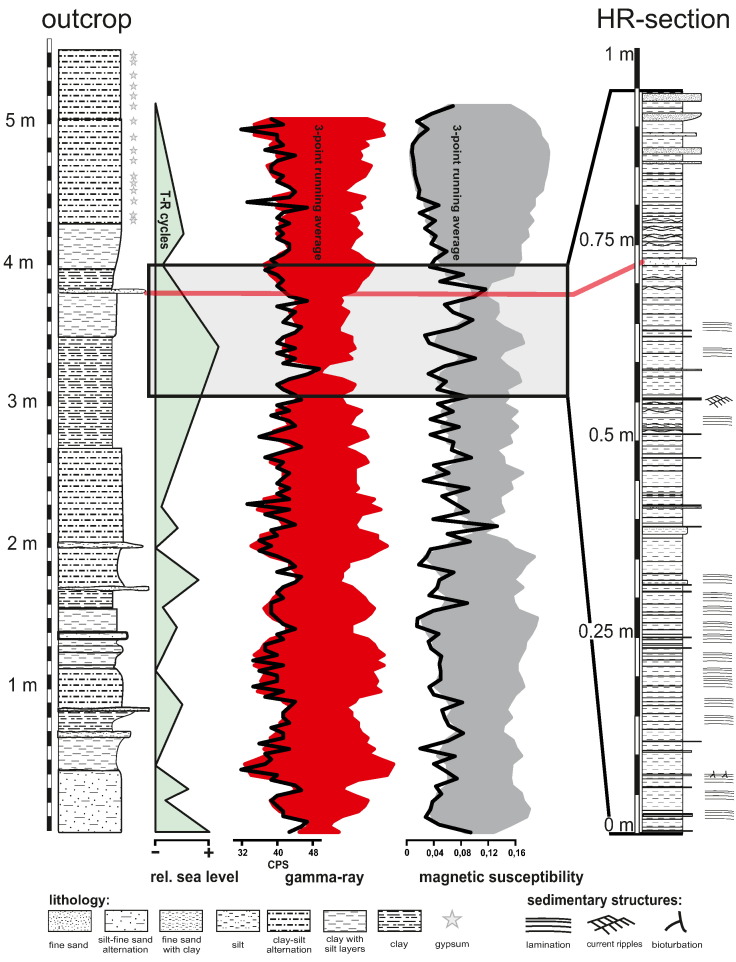
Lithologs of the 5.5-metre-outcrop and the high-resolution section. Position of the HR-section within the 5.5-metre-section is indicated by the grey rectangle. Gamma-ray emission and magnetic susceptibility logs are shown as black lines. A three point running mean was calculated for both measurements and is shown as mirrored column for both measurements. The gamma-ray log was used to reconstruct changes in relative sea level.

The exposed sedimentary succession in the clay pit has an overall thickness of 5.5 m and consists of finely laminated blue-grey to green-grey clays with intercalations of fine sand and silt lenses (Fig. 3). Lamination varies on a sub-millimetre to centimetre scale. The intercalated silt and fine sand layers reach a thickness of up to 50 mm and decrease in frequency and thickness towards the top of the succession and frequently show well-preserved current ripples. The sediment is mostly free of bioturbation as well as macrofossils. Natural gamma radiation and magnetic susceptibility were logged continuously for the whole outcrop, using a portable scintillation counter (Heger–Breitband–Gammasonde) and a portable magnetic susceptibility meter (Exploranium KT-9), respectively (Fig. 3).

Starting at 3.06 m above the base of the succession a high-resolution section was studied with an overall thickness of 940.5 mm (Fig. 3). 100 layers were logged and sampled by completely removing a substantial portion (> 300 g) of each layer. The top of each newly exposed layer was thoroughly cleaned of debris before sampling. This top down approach was applied because it allowed clean retrieval of all samples. For subsequent analyses a portion of each sample was extracted after thoroughly homogenising the sediment from the respective layer.

The high-resolution section is situated in a relatively undisturbed sequence of finely laminated clays with some intercalations of fine sand and silt. The intercalations increase in frequency and thickness in the topmost part of the section and some show current ripples. Weak bioturbation is only present in two successive layers of laminated clays near the base of the section. Otherwise a clear lamination dominates the lower half of the section but becomes progressively less prominent to the top. Additionally, the layers become less clearly defined and wavy. This trend is coupled with a decrease in gamma ray intensity and magnetic susceptibility in the topmost part of the high-resolution section (Fig. 3).

### 3.2 Sample preparation

Approximately 10 g of each of the 100 samples was powdered and 0.1–0.15 g was analysed using a LECO CS230 for wt.% of total carbon (TC), sulphur and total organic carbon (TOC). Based on the TC and TOC the total inorganic carbon (TIC = TC − TOC) content was calculated, which was then used to compute the calcite equivalent carbonate content using the stoichiometric formula (8.34 ∗ TIC) (Stax and Stein, 1995, Grunert et al., 2010a). Additionally, the ratio of organic carbon to sulphur (C/S-ratio) was calculated (Berner and Raiswell, 1984).

Smear slides were prepared for analysis of calcareous nannofossil assemblages following the standard preparation methods of Bown and Young (1998). We opted to leave the sediment untreated in order to preserve the original composition of the assemblages. To facilitate disaggregation they were ultrasonicated for 5 s before transfer of the suspension onto the coverslip. The slides were mounted using Eucit® and studied with a standard light microscope at a magnification of 1000 × under parallel and crossed nicols. Approximately 300 specimens were counted in each sample. The assemblages were then analysed with respect to their similarity/dissimilarity to each other, as well as shifts in abundances of taxa over time.

### 3.3 Taxonomic remarks

The taxonomy used herein is largely based on Perch-Nielsen, 1985a, Perch-Nielsen, 1985b, Young (1998), Varol (1998) and Burnett (1998), supplemented by the Handbook of Calcareous Nannoplankton 1–5 (Aubry, 1984, Aubry, 1988, Aubry, 1989, Aubry, 1990, Aubry, 1999) and the Nannotax website (Young et al., 2013). The revised taxonomy for the Paratethys by Galović and Young (2012) was also considered.

Specimens were identified to the species level wherever possible. Subsequently all identified taxa were grouped according to their recorded stratigraphic range, in order to create a stratigraphic framework and quantify the amount of allochthonous taxa present in the assemblage. The autochthonous taxa were then analysed using multivariate statistics (cluster-analyses and nMDS) to reconstruct the palaeoenvironment. The use of the terms allochthonous and autochthonous follows the guidelines of Martin (1999) regarding fossil assemblages.

The genus *Reticulofenestra* is a morphologically diverse and somewhat poorly constrained coccolith taxon ranging from Eocene to Recent (Young, 1998). For this study the general distinction of Young (1998) based on the placolith size was used: *Reticulofenestra minuta* < 3 μm, *Reticulofenestra haqii* 3–5 μm, *Reticulofenestra pseudoumbilicus* > 5 μm. The frequently applied distinction between medium-sized reticulofenestrids (3–5 μm) with open and closed central areas (*R. haqii* (open) versus *Reticulofenestra antarctica* (closed)) (Wade and Bown, 2006) was not used because of the high amount of reworked and poorly preserved specimens. Other allochthonous taxa of the genus *Reticulofenestra* from the Palaeogene were identified following the morphotype concept of Varol (1998).

### 3.4 Statistical treatment and analyses

The number of specimens per field of view in the light microscope was used as a rough indicator of the total abundance of calcareous nannofossils in the samples. The arithmetic mean as well as the standard deviation for the collected datasets were calculated using the percentages of taxa within each sample. The Shannon–Wiener diversity index, the dominance index and the species evenness (Shannon and Weaver, 1948, Sokal and Rohlf, 1995, Wade and Bown, 2006) were calculated using the statistics software PAST® (Hammer et al., 2001).

In order to ensure the normal distribution of the relative abundances, the studied dataset was transformed using the arcsine transformation as outlined in Sokal and Rohlf (1995). The reason for using this transformation method was to generate a well-suited dataset for subsequent statistical treatments.

In order to verify that allochthonous specimens of long ranging taxa do not obscure the ecological signal of the autochthonous assemblage an R-mode analysis was performed. For this analysis both cluster analysis (Ward's-method) and non-metric multidimensional scaling (nMDS; Euclidean similarity measure) were applied (Hammer and Harper, 2006).

To determine assemblages a Q-mode cluster analysis was performed using Ward's-method (Ward, 1963, Hammer and Harper, 2006). To assess which taxa are primarily responsible for the differences between clusters, a similarity percentage analysis (SIMPER) was performed using the Bray–Curtis similarity measure (Bray and Curtis, 1957, Clarke, 1993, Hammer and Harper, 2006).

## 4 Results

### 4.1 Nannoplankton taphonomy

Of the 124 recorded taxa only 10 of them occur with a mean > 1%. These represent an average of 78.64% (σ = 4.61) of the total assemblage. In decreasing abundances the taxa are: *Coccolithus pelagicus* (34.52%; σ = 4.91), *R. minuta* (12.48%; σ = 4.95), *Cyclicargolithus floridanus* (8.57%; σ = 3.23), *Watznaueria barnesae* (8.47%; σ = 3.07), *R. haqii* (5.76%; σ = 2.51), *R. pseudoumbilicus* (2.18%, σ = 1.19), *Reticulofenestra bisecta* (2.04%; σ = 1.14), *Prediscosphaera cretacea* (1.72%; σ = 0.91), *Cyclagelosphaera reinhardtii* (1.72%; σ = 1.17) and *Lanternithus minutus* (1.18%; σ = 0.85).

The remaining 114 taxa (< 1%) represent on average 21.36% (σ = 4.61) of the total assemblage. Some accessory taxa, e.g., *Braarudosphaera bigelowii*, *Helicosphaera carteri* and *Sphenolithus moriformis*, show elevated abundances in certain samples.

Of all taxa recorded, only 24 occur within Zone MNN4a (see taxonomic list in the supplement). These represent an average of 67.86% (σ = 5.33) of the total assemblage. Some of them display a wide stratigraphic range and thus likely contain specimens that are reworked from Paleocene to Eocene sediments (see below). Such taxa are *C. pelagicus*, *C. floridanus*, as well as *S. moriformis* and *R. minuta*. Since in such cases allochthonous specimens could not be readily distinguished from autochthonous ones all specimens belonging to these taxa were considered to be part of the autochthonous assemblage. However, subsequent analyses indicate that the ecological signal of these taxa is largely unaffected by this partial contamination.

**Fig. 4:**
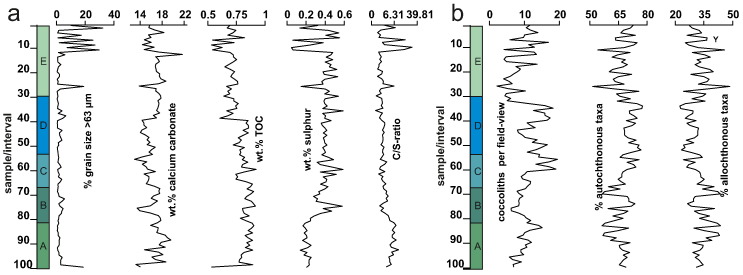
Sedimentological (% of grains > 63 μm) and geochemical data (amount of carbonate, organic carbon and sulphur as well as the C/S-ratio) of the high-resolution section. Coccolith abundances are shown as coccoliths encountered per field of view under the light microscope and the abundance of clearly autochthonous and allochthonous specimens in %. The coloured and labelled columns indicate Intervals A–E.

100 clearly allochthonous taxa were found: 41 taxa can be attributed to the Palaeogene, mainly Paleocene and Eocene and 59 were clearly Cretaceous (mainly Campanian to Maastrichtian). Palaeogene taxa include *R. bisecta*, *Reticulofenestra umbilica*, *L. minutus*, *Zygrhablithus bijugatus*, as well as *Cyclicargolithus luminis*. The Cretaceous assemblage is dominated by *W. barnesae*, *P. cretacea*, *C. reinhardtii*, *Micula staurophora* and *Micula decussata*. All allochthonous taxa were grouped together for subsequent statistical treatments as a proxy for terrigenous input (Fig. 4).

### 4.2 Nannoplankton abundance and diversity

Within the autochthonous assemblage only seven taxa exhibit an average abundance of > 1% and represent on average 96.55% (σ = 1.67) of the total autochthonous assemblage. The other 3.45% (σ = 1.67) are represented by the remaining 17 taxa. Normalized to 100% the seven most abundant taxa of the autochthonous assemblage are: *C. pelagicus* (51.16%; σ = 6.51), *R. minuta* (18.45%; σ = 7.20), *C. floridanus* (12.60%; σ = 4.34), *R. haqii* (8.46%; σ = 3.51), *R. pseudoumbilicus* (3.26%; σ = 1.81), *Coronosphaera mediterranea* (1.40%; σ = 1.14) and *S. moriformis* (1.22%; σ = 0.79).

**Fig. 5:**
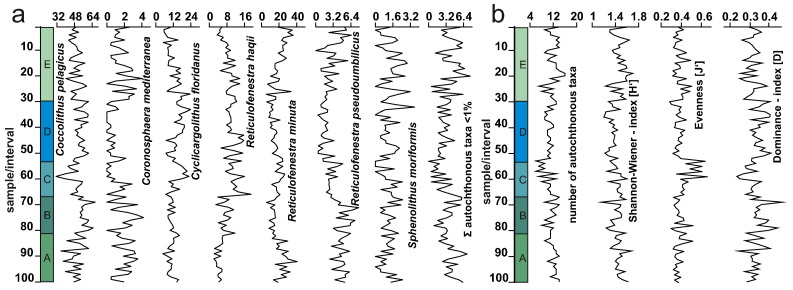
Relative abundances of autochthonous coccolith taxa > 1% as well as the abundance of autochthonous taxa < 1%, normalized to 100%, within each sample of the high-resolution section. Diversity indices show the amount of autochthonous taxa, the Shannon–Wiener index, species evenness and the dominance index of the studied samples. The coloured and labelled columns indicate Intervals A–E.

The maximum number of taxa present in a single sample is 16 (samples 20 and 19), the minimum is 6 (samples 53 and 57). On average 11 (σ = 2.03) autochthonous species are present in each sample (Fig. 5). The Shannon–Wiener diversity index (H′) ranges from 1.195 to 1.738 with an average of 1.47 (σ = 0.11). The average species evenness (J′) is 0.40 (σ = 0.07) with J′max = 0.66 and J′min = 0.20. The dominance index (D) has an average value of D = 0.33 (σ = 0.04) with Dmax = 0.47 and Dmin = 0.24 (Fig. 5).

### 4.3 Nannoplankton assemblages

**Fig. 6:**
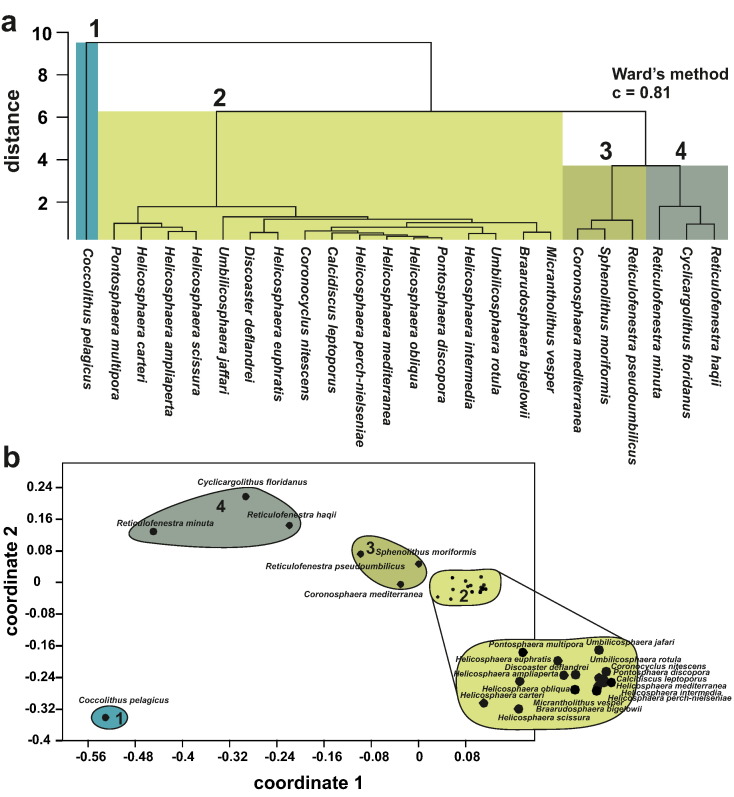
(a) R-mode cluster analysis of arcsine-root method transformed abundances of all encountered autochthonous taxa using Ward's method. Clusters, indicated by the coloured areas, represent taxa with similar ecological preferences. (b) Non-metric multidimensional scaling analysis of the autochthonous assemblage. Arrows are used to indicate ecological factors, which may determine the distribution of the taxa within the plot.

The cluster analyses including all autochthonous taxa reveal four clusters (Fig. 6a), which were used to classify the autochthonous assemblages: **Assemblage 1** corresponds to a single species, *C. pelagicus*. **Assemblage 2** contains 17 taxa all of which have a mean abundance of < 2% including all species of the genera *Braarudosphaera*, *Calcidiscus*, *Coronocyclus*, *Discoaster*, *Helicosphaera*, *Micrantholithus*, *Pontosphaera* and *Umbilicosphaera*. **Assemblage 3** includes *C. mediterranea*, *R. pseudoumbilicus* and *S. moriformis*. **Assemblage 4** contains *C. floridanus*, *R. haqii* and *R. minuta*.

The nMDS supports the grouping into four clusters. While clusters 1 and 4 are clearly separated in the nMDS, clusters 2 and 3 plot in close proximity to each other (Fig. 6b). While cluster analysis indicates a closer relationship between clusters 3 and 4, the relative proximity of clusters 2 and 3 within the nMDS may indicate that cluster 3 can be seen as transitional between clusters 2 and 4, also in terms of ecological preferences.

### 4.4 Nannoplankton taphogroups

**Fig. 7:**
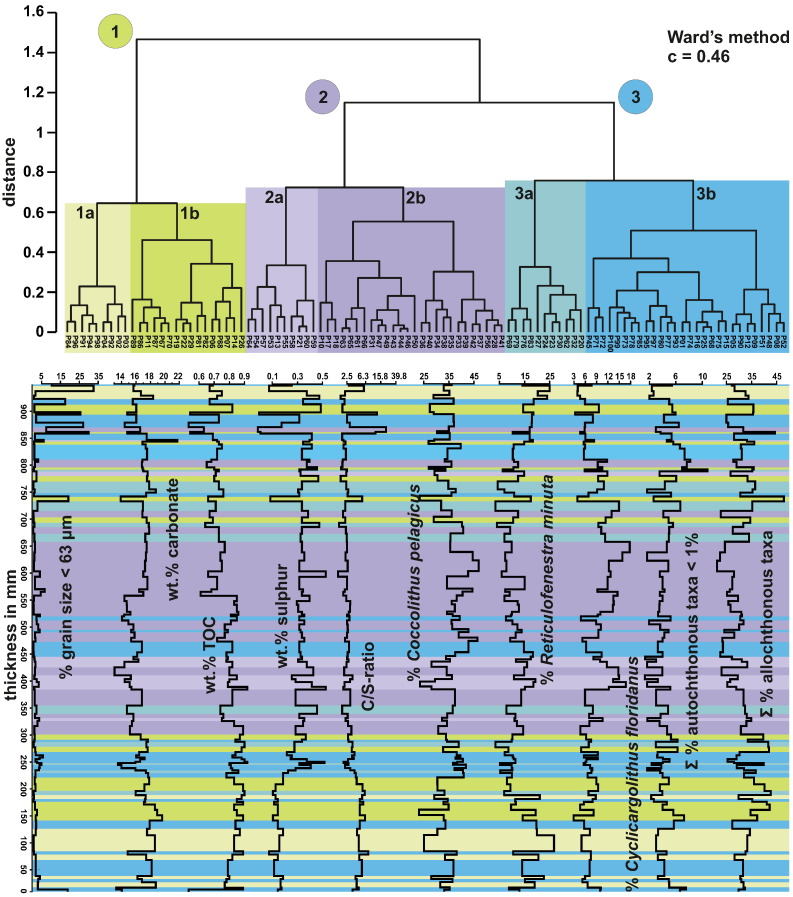
Q-mode cluster analysis of arcsine-root method transformed abundances of the five key taxa (*Coccolithus pelagicus*, *Cyclicargolithus floridanus*, *Reticulofenestra haqii*, *Reticulofenestra minuta*, *Reticulofenestra pseudoumbilicus*) combined with the abundance of taxa < 1% and the amount of allochthonous taxa. Significant clusters were numbered (1–3) with their sub-assemblages labelled *a* and *b* respectively, and assigned colours. The colour-code was applied to each layer according to their cluster-affiliation within the section and plotted in conjunction with sedimentological, geochemical and coccolith abundance data. The thickness of the coloured bars indicates the thickness and positions of each sampled layer within the section.

Both autochthonous and allochthonous taxa were used to define taphonomic associations labelled as taphogroups (Fig. 7).

Autochthonous taxa with a mean of < 2% (normalised to 100%) were grouped together based on the results of the R-mode nMDS (Fig. 6b). Taxa with a mean > 2% (*C. pelagicus*, *C. floridanus*, *R. haqii*, *R. minuta*, *R. pseudoumbilicus*) were left ungrouped to reflect their strong influence on the assemblage. The allochthonous specimens were also grouped to reflect the amount of terrigenous influx in each sample. The analysis resulted in three clusters with two sub-clusters each (Fig. 7).

SIMPER analysis (Table 1) shows that the calcareous nannofossil taphocoenoses are generally defined by: (1) the amount of allochthonous taxa; (2) the differences in the abundances of *C. pelagicus* and *C. floridanus* compared to *R. haqii*, *R. minuta* and *R. pseudoumbilicus*; and (3) the abundance of accessory autochthonous taxa in the autochthonous assemblages.

**Table 1:** SIMPER analysis using Bray–Curtis similarity showing the contribution (in percent) of all used groups of calcareous nannofossils to their respective taphogroups (TG) 1a,b; 2a,b; 3a,b.

Group	Contribution	Cumulative %	TG 1a	TG 1b	TG 2a	TG 2b	TG 3a	TG 3b
Allochthonous taxa	3.267	22.89	31.90	40.00	30.80	28.80	38.00	30.00
*Reticulofenestra minuta*	3.000	43.90	22.00	12.50	15.20	10.90	5.45	12.60
*Coccolithus pelagicus*	2.941	64.51	30.10	29.30	30.10	37.40	36.00	37.20
*Cyclicargolithus floridanus*	1.897	77.79	5.95	6.83	11.80	10.70	8.78	7.22
*Reticulofenestra haqii*	1.493	88.25	2.81	4.17	7.80	7.66	5.78	5.11
Autochthonous taxa < 1%	0.955	94.94	3.94	4.73	2.18	3.47	4.14	4.92
*Reticulofenestra pseudoumbilicus*	0.722	100.00	3.22	2.55	1.99	1.05	1.79	2.90

**Taphogroup 1** occurs prominently in the lowermost and uppermost parts of the section (Fig. 7) and is characterised by a relatively high amount of allochthonous taxa (Table 1). This is coupled with a high contribution of *R. minuta* and low abundances of *C. pelagicus* and *C. floridanus*. The two sub-groups (1a & 1b) are defined by their difference in the abundance of allochthonous taxa as well as small reticulofenestrids. Sub-group 1a shows the highest amount of *R. minuta* whereas 1b that of allochthonous taxa. All other taxa remain relatively constant.

**Taphogroup 2**, based on cluster 2, dominates the middle part of the section (Fig. 7). It has the highest amount of *C. pelagicus* and *C. floridanus*. It exhibits very low amounts of allochthonous taxa, indicating a decrease in terrigenous influx in those samples. The sub-groups (2a & 2b) are generally defined by a shift in the amount of *C. pelagicus* (highest in 2a) and *C. floridanus* (highest in 2b).

**Taphogroup 3** shows consistently high abundances of *C. pelagicus* with moderately high to low abundances of other autochthonous taxa such as *C. floridanus* and *R. minuta*. Sub-group 3a exhibits very low abundances of *R. minuta*, while its abundance is quite high in sub-group 3b. The amount of allochthonous taxa in sub-group 3a is higher (Table 1).

**Fig. 8:**
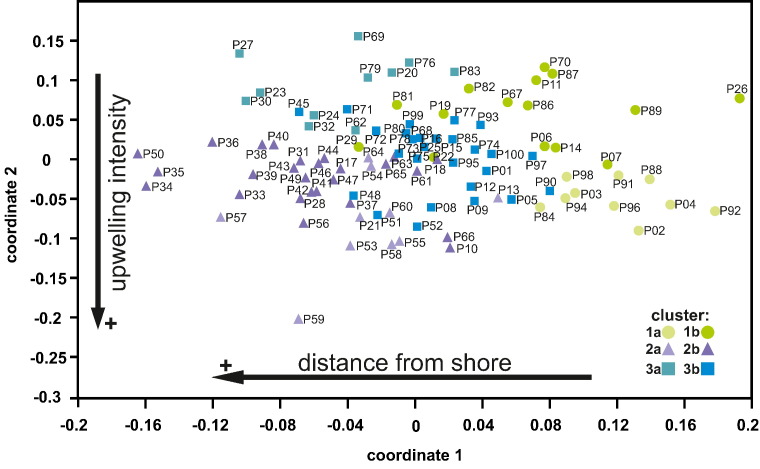
Q-mode non-metric multidimensional scaling analysis of the high-resolution section. Colours indicate the cluster affiliated with each sample. Arrows indicate the possible ecological conditions that determined the distribution of the samples within the plot.

The nMDS using the Bray–Curtis dissimilarity measure (Bray and Curtis, 1957, Hammer and Harper, 2006) revealed a clear trend of the samples, grouping them in accordance with the clusters (Fig. 8). The fact that samples do not form distinct groups in the nMDS indicates a gradual transition between the taphogroups defined by the cluster analysis.

## 5 Discussion

Palaeoenvironmental reconstructions indicate that the easternmost part of the NAFB in the area around Laa an der Thaya represents a marine outer neritic during the early Karpatian around 17 Ma (Spezzaferri and Ćorić, 2001). At this time the western part of the NAFB was already a brackish water environment with eastwards encroaching freshwater sedimentation (Kuhlemann and Kempf, 2002).

In the 5.5-metre-succession frequent fluctuations in the sedimentation regime occur as indicated by both gamma ray and magnetic susceptibility logs. The logs indicate a waxing and waning of coarser sediments in a depositional succession dominated by clays and silts. These changes led to the occurrences of funnel and bell shaped patterns within a typical saw-tooth pattern in the datasets interpreted as changes in the relative sea level during deposition (Fig. 3).

### 5.1 Palaeoecological interpretation

Statistical analyses of the autochthonous coccoliths resulted in the differentiation of four assemblages, with each assemblage containing taxa with distinct ecological preferences (Fig. 6a):

**Assemblage 1** is formed solely by *C. pelagicus*. Today, one sub-species, *C. pelagicus* ssp. *pelagicus* (< 10 μm), is the most common in the northern regions of the Atlantic Ocean (McIntyre and Bé, 1967, Ziveri et al., 2004). In contrast, the larger (> 10 μm) sub-species *C. pelagicus* ssp. *braarudii* frequently occurs in areas of increased coastal upwelling such as the southern and northern Benguela upwelling regimes (Giraudeau et al., 1993, Giraudeau and Rogers, 1994) and the Portuguese upwelling front, where it is specifically associated with zones of moderate upwelling intensity (Cachão and Moita, 2000). It shows optimum growth conditions in water masses with SST-ranges from 2 to 12 °C (Okada and McIntyre, 1979). Outside its optimum conditions the species is still well represented in cold water masses with temperatures as low as − 1.7 °C and as high as 15 °C (Okada and McIntyre, 1979, Winter et al., 1994). Cachão and Moita (2000) extended the maximum temperature range of *C. pelagicus* even up to 16–18 °C. Apart from temperature, *C. pelagicus* also shows an affinity for nutrient-rich waters (McIntyre and Bé, 1967, Roth, 1994, Cachão and Moita, 2000). Additionally, Silva et al. (2008) observed that *C. pelagicus* is able to flourish in waters with a considerable range of salinity (26.9–36.0‰). While the smaller subspecies dominates the assemblage in the section studied herein the ecological preferences of both sub-species were considered based on the palaeogeographic setting of the studied section.

**Assemblage 2** contains all taxa < 2% and can be interpreted as an assemblage that mainly consists of taxa preferring temperate to warm water with varying dependencies on nutrient availability. The tight grouping is mostly due to the very low abundances of those taxa, which conversely also reflects their very similar ecological preferences. Notable palaeoecological indicators such as *Calcidiscus leptoporus*, *Discoaster deflandrei*, *Umbilicosphaera jafari* and *Umbilicosphaera rotula* reflect the strong preference of this assemblage for warmer SSTs and/or lower nutrient concentrations (Okada and Honjo, 1973, Haq, 1980, Winter et al., 1994).

Only the smaller (< 5 μm) morphotype of *C. leptoporus* (Sáez et al., 2003, Quinn et al., 2004) occurs in the section. Based on the evolutionary history of the different morphotypes of *C. leptoporus* (Knappertsbusch, 2000) and the still rather unclear ecology of extant populations of the smaller morphotype (Quinn et al., 2004, Ziveri et al., 2004) an accurate interpretation of the ecological preferences of *C. leptoporus* is not possible. Nevertheless, more recent studies found *C. leptoporus* (sensu lato) in the cold (~ 14–15 °C) waters of Lisbon Bay during summer (Cachão and Moita, 2000, Cachão et al., 2000) and Silva et al. (2008) consider *C. leptoporus* as a tracer for the influx of subtropical surface offshore waters during winter and conclude that *C. leptoporus* is an indicator for periods of lower productivity and a member of a typical transitional assemblage from colder to warmer coccolithophore communities.

Similarly, the presence of the genus *Helicosphaera* in this assemblage indicates hemipelagic settings with a possible occurrence of upwelling (Perch-Nielsen, 1985b, Ziveri et al., 2004). *H. carteri* is the most abundant species of the genus in the studied section. It is considered a thermophilic coastal taxon in modern oceans (Okada and McIntyre, 1979, Ziveri et al., 2004) and indicates moderate nutrient availability as well as moderate turbulence in the water column (Roth and Berger, 1975, Giraudeau and Rogers, 1994, Flores et al., 1995, Negri and Villa, 2000). Other helicosphaerids in the assemblage are: *Helicosphaera ampliaperta*, *Helicosphaera euphratis*, *Helicosphaera mediterranea*, and *Helicosphaera scissura*. All are generally considered to prefer warm to temperate waters and increased nutrient availability (Perch-Nielsen, 1985b, Ziveri et al., 2004). *Pontosphaera discopora* and *Pontosphaera multipora* are generally described as preferring shelf areas, while being uncommon in open ocean settings (Perch-Nielsen, 1985b).

*B. bigelowii* and the closely related *Micrantholithus vesper* (Street and Bown, 2000, Bown, 2005) show rather ambiguous ecological preferences (see Aubry, 2013 for extensive discussion). Nevertheless, the species often bloom in waters of low salinity or even brackish conditions in coastal areas (Peleo-Alampay et al., 1999) and appear to also thrive under highly eutrophic conditions (Cunha and Shimabukuro, 1997) with a strong influx of terrigenous material (Svabenicka, 1999). Since they also occur in the open ocean (Siesser et al., 1992, Peleo-Alampay et al., 1999, Kelly et al., 2003) and especially during times of reduced competition and raised nutrient availability (Thierstein et al., 2004), both species cannot be necessarily linked to neritic environments, but rather to periods of high nutrient availability and/or increased environmental stress (Wade and Bown, 2006). Based on the palaeogeographical setting of the section especially *B. bigelowii* can thus be regarded as an indicator of environmental stress most likely related to lowered salinity or increased terrigenous influx, caused by an increased proximity to the shore.

**Assemblage 3** consists of three taxa (*R. pseudoumbilicus*, *C. mediterranea*, *S. moriformis*) that cannot be easily linked according to their preferences for water temperature or nutrient availability. *R. pseudoumbilicus* is indicative of high nutrient levels with no particular preferences for water temperature (Lohmann and Carlson, 1981). *C. mediterranea* occurs in modern temperate to warm waters, with marked spikes during times between upwelling and downwelling pulses at a moderate distance from the shore (Silva et al., 2008). Similarly, while typically being a taxon preferring warm open marine conditions, *S. moriformis* seems to be able to still prosper in shallower coastal areas and also times of considerable environmental stress (Wade and Bown, 2006). Assemblage 3 can thus be interpreted to reflect a preference for shallow waters and near-shore environments with generally warm to temperate SSTs and a tolerance for varying nutrient availability; it is considered to be transitional between Assemblages 2 and 4.

**Assemblage 4** includes small and medium-sized reticulofenestrids (*R. haqii*, *R. minuta*) as well as *C. floridanus* and can be interpreted as an assemblage that is indicative of nutrient-rich waters without strong fluctuations in temperature, nutrient availability and surface water turbulence. *R. minuta* in particular is often associated with an increased availability of terrigenous nutrients and stable water conditions (Haq, 1980, Wade and Bown, 2006). Wei and Wise (1990) consider *C. floridanus* an indicator for temperate waters. Aubry (1992) suggested that the distribution of *C. floridanus* is not necessarily only an expression of temperature, but could also reflect high nutrient availability.

Based on the ecological preferences of the considered taxa the cluster distribution in the nMDS (Fig. 6b) can be interpreted in the following way: Coordinate 1 of the nMDS correlates positively to calcareous nannoplankton taxa able to thrive in waters in a closer proximity to the shore. Coordinate 2 displays a strong negative correlation with the preferences for upwelling conditions. Temperature was defined as a third ecological factor in the nMDS ranging between coordinate axes 1 and 2. The temperature gradient shows a positive correlation with both coordinates, placing taxa that prefer cooler water conditions on the lower left side and taxa that prefer warmer SSTs on the upper right side. These results indicate that a primary ecological signal is preserved in the autochthonous assemblages, despite the fact that certain taxa are likely contaminated by allochthonous specimens.

The three taphogroups (Fig. 7), with two sub-groups each, reflect small-scale changes in palaeoenvironmental conditions:

Taphogroup 1 The high amount of *R. minuta* in sub-group 1a indicates high nutrient levels of terrigenous origin. The high amount of allochthonous taxa in sub-group 1b can be correlated with high terrigenous influx. Low amounts of the typical open water species *C. floridanus* support the assumption that this group occupies a position relatively close to the shore. Similarly, the elevated content of warm water taxa as well as the low content of *C. pelagicus* points towards generally warmer SSTs compared to the other two groups. In summary, Taphogroup 1 is interpreted as being indicative of high terrigenous influx and a close proximity to the shore.
Taphogroup 2 High amounts of the open ocean species *C. floridanus* (sub-group 2a) and *C. pelagicus* (sub-group 2b) indicate that Taphogroup 2 can be correlated with the greatest distance from the shore. This is also supported by a low content of warm water and allochthonous taxa. Sub-group 2a was associated with waters showing reduced water turbulence, based on the higher amounts of *C. floridanus*. Sub-taphogroup 2b was adapted to upwelling conditions based on the preference of *C. pelagicus* for cool nutrient-rich waters.
Taphogroup 3 This group can be interpreted as a transitional group between groups 1 and 2. This assumption is based on high abundances of *C. pelagicus* that are coupled with moderately low to very low abundances of typical warm water taxa. *C. floridanus* is also low in abundance. High contents of *R. minuta* (sub-group 3b) and allochthonous taxa (sub-group 3a) indicate terrigenous influx and high nutrient availability similar to Taphogroup 1. Higher amounts of *C. pelagicus* in both sub-groups indicate colder SSTs comparable to sub-group 2b.

The nMDS plot shows a gradual progression from samples dominated by cool SSTs and upwelling conditions to samples that are dominated by terrigenous influx and relatively warmer SSTs. The samples do not form separated clusters, but a clear trend is still apparent. Coordinate 1 can be thus correlated with the proximity to the shore, while Coordinate 2 is negatively correlated with upwelling intensity (Fig. 8).

### 5.2 Palaeoenvironmental model

The regime under which the studied section was deposited can be considered as a marine near-shore environment in the eastern part of the NAFB. It is characterised by calcareous nannofossil assemblages that point towards cool to temperate SSTs and high nutrient availability associated with terrigenous input and wind-driven upwelling. Geochemical data and the largely absent bioturbation indicate anoxic to dysoxic bottom water conditions. Previous studies (Spezzaferri and Ćorić, 2001, Spezzaferri et al., 2002) further indicated a relative proximity to the shore with water depths not exceeding 200 m based on studied assemblages of benthic foraminifers.

**Fig. 9:**
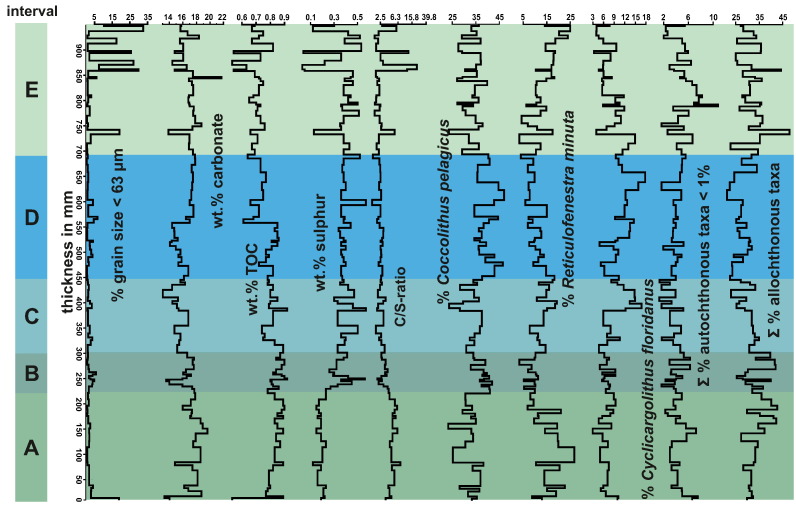
Graphical representation the five described intervals (Interval A–E), as a labelled column. The intervals were additionally plotted in conjunction with key sedimentological, geochemical and coccolith abundance data (see Fig. 6) transformed to reflect the thickness of each layer.

While the overall conditions remain more or less stable during the deposition of the 940-mm-thick section, the distribution of the defined assemblages and taphogroups and to a lesser degree changes in geochemical (see below) and sedimentological proxies clearly point to small-scale palaeoenvironmental variations. These data allow a differentiation of the section into five intervals (A–E; Fig. 9):

Interval A Shallow neritic fresh water influenced environment
Samples 100–81 (0–212 mm) (Fig. 9)
Lithology calcareous silty clay
Taphogroups 1a, 1b, 3b (3a) (Fig. 7)

Interval A is the most notable for its low content in sulphur compared to all other intervals in the section. The low sulphur content coupled with a continuously high carbonate content point towards much better oxygenated bottom waters compared to the rest of the section. Higher contents of TOC point to higher fertility levels (Berner and Raiswell, 1984, Kuypers et al., 2002, Twichell et al., 2002).

The nannoplankton assemblages predominantly consist of *C. pelagicus* and *R. minuta* (Fig. 5, Fig. 7, Fig. 9). Both indicate high primary productivity under eutrophic conditions. The elevated content of small reticulofenestrids points to strong influx of terrigenous material and a relative proximity to the shore (Haq, 1980, Aubry, 1992, Flores et al., 1995, Wade and Bown, 2006). This is further supported by high amounts of allochthonous taxa in this interval, indicating increased continental runoff (Fig. 9).

*R. minuta* is considered to thrive during times of strongly fluctuating environmental conditions (Wade and Bown, 2006), so the intercalation of Taphogroups 1 and 3 is most likely indicative of increased continental runoff, explaining changes in the abundance of *R. minuta* and *C. pelagicus*. Layers characterised by Taphogroups 1a and 1b and thus higher amounts of *R. minuta* or allochthonous taxa indicate strong terrigenous influx with raised nutrient availability possibly coupled with an increased stratification of water masses (Ćorić and Hohenegger, 2008). This may further point to repeated input of surface bound fresh water transporting high amounts of reworked Cretaceous and Palaeogene nannoplankton taxa. Hence, the alternation between Taphogroups 1 and 3 can be roughly correlated with a variability of precipitation in the hinterland. Low levels of sulphur coupled with continuously high amounts of carbonate and organic carbon (Fig. 4, Fig. 9), indicate stable, at least partly oxygenated, bottom-water conditions. Such conditions could also account for the sporadic bioturbation in the lower part of the high-resolution section (Fig. 3). Berner and Raiswell (1984) point out that levels of organic carbon below 1 wt.% are not suitable for a reliable interpretation of the C/S-ratio. However, the general trend of the C/S-ratio in the section still reflects the strong terrigenous influence in Interval A. Raised levels (2.83–7.41) would at least indicate slightly brackish conditions.

Interval B Transition from fresh water influence to more stable marine conditions
Samples 80–67 (212–292 mm) (Fig. 9)
Lithology calcareous silty clay
Taphogroups 3b (3a & 1b) (Fig. 7)

Interval B marks the onset of more dysoxic to anoxic bottom water conditions that are indicated by a slight increase in sulphur (Berner, 1981, Maynard, 1982, Berner and Raiswell, 1984). The sediment is finely laminated in this part of the section. The C/S-ratio decreases to typical marine conditions pointing towards higher salinity levels and the waning of freshwater influx (Fig. 4, Fig. 7, Fig. 9). The decrease in bottom water oxygenation coupled with a decrease in influx of terrigenous material and freshwater, is also expressed by a decrease in small reticulofenestrids (Haq, 1980, Aubry, 1992, Flores et al., 1995, Wade and Bown, 2006). This decrease further points towards a decrease in precipitation in the hinterland or a slight rise in sea level.

This interval shows a dominance of Taphogroup 3. The increase in *C. pelagicus* may indicate the possible establishment of a more stable wind-driven upwelling cell in the lower part. Lower amounts of reticulofenestrids as well as allochthonous taxa indicate a decrease of terrigenous influx (McIntyre and Bé, 1967, Aubry, 1992, Cachão and Moita, 2000, Persico and Villa, 2004). The increase in *C. pelagicus* goes hand in hand with a small decrease in the overall abundance of taxa compared to Interval A (Fig. 5, Fig. 9), reflecting an increase in cool nutrient-rich water masses that are no longer influenced by fluctuations in salinity. An ongoing decrease in the amount of allochthonous calcareous nannofossils further points towards a decrease in riverine outflow.

The lower part of Interval B marks the first increase in upwelling conditions, which favoured *C. pelagicus* and primary productivity also increased. Bottom water conditions, however, remained more or less stable and stagnant. The top of Interval B is characterised by a short re-establishment of conditions similar to Interval A (samples 70–67; Fig. 7, Fig. 9).

Interval C Eutrophic marine conditions
Samples 66–53 (292–431 mm) (Fig. 9)
Lithology calcareous silty clay
Taphogroups 2a, 2b (3a) (Fig. 7)

Interval C marks the first occurrence of Taphogroup 2. *C. pelagicus* decreases slightly towards the top and *C. floridanus* increases strongly in the uppermost part (sub-group 2a; Fig. 7, Fig. 9). Reticulofenestrids also increase towards the top. The decrease of *C. pelagicus* coupled with increases in small and medium-sized reticulofenestrids and *C. floridanus* indicates a reduction in water turbulence and upwelling (Fig. 5, Fig. 7, Fig. 9). Sulphur levels fluctuate but are similar to Interval B. The amount of TOC decreases slightly towards the top. The C/S-ratio stays consistently at normal marine values, ranging from 2.58 to 1.5 (Fig. 4, Fig. 7, Fig. 9). More stable conditions are also supported by the high species evenness of the assemblage (Fig. 5).

Interval C can thus be interpreted to reflect more open marine conditions linked to a decrease in freshwater influx. The latter is also indicated by a continued decrease in allochthonous taxa. A decrease in *C. pelagicus* further indicates decreases in upwelling conditions or a slight increase in temperature. A shift in the surface water conditions could either point towards a decrease in precipitation, a shift in current direction, or a slight rise in sea level.

Interval D Eutrophic marine conditions with upwelling
Lithology calcareous silty clay
Samples 52–30 (431–684 mm) (Fig. 9)
Taphogroups 2b (3b & 3a) (Fig. 7)

Interval D can be described as the most stable interval mainly consisting of Taphogroup 2b. The lower part is characterised by a short appearance of Taphogroup 3b, while the uppermost part shows a short appearance of Taphogroup 3a (Fig. 7). A sharp increase in the abundance of *C. pelagicus* is coupled with a decrease of all other autochthonous taxa in the assemblage. Allochthonous taxa are rare (Fig. 9) and diversity is the lowest in this part of the section (Fig. 5). The generally high amount of *C. pelagicus* is still subject to fluctuations, indicating changes in upwelling intensity. These fluctuations are similarly reflected in diversity. While both Shannon–Wiener-index and species evenness show lower values during suspected upwelling, the dominance index clearly increases (Fig. 5).

Increased primary productivity is also reflected by a high amount of sulphur (Fig. 9). The content of sulphur further points towards continued low oxygenation on the ocean floor (Berner, 1981, Maynard, 1982, Berner and Raiswell, 1984). The assumption of a raised primary productivity during Interval D is further supported by an increase in overall coccolith abundance (Fig. 4).

It can be assumed that an increase in abundance of *C. pelagicus* coupled with an increase in overall abundance of coccoliths indicates a bloom of *C. pelagicus* (Fig. 4, Fig. 5). Hence, Interval D may be assumed to reflect wind driven upwelling conditions. Dysoxic conditions continue at the ocean floor, as indicated by high sulphur contents and the absence of bioturbation. The C/S-ratio exhibits typical values for normal marine conditions throughout Interval D (Fig. 8).

Interval E Decreasing sea level with high terrigenous influx
Lithology calcareous silty clay with episodic intercalation of sand
Samples 29–01 (684–940.5 mm) (Fig. 9)
Taphogroups 1–5 (Fig. 7)

Interval E displays a rather different distribution of taphogroups compared to the previous four intervals. It shows strong fluctuations of the nannoplankton assemblage as well as well-correlated changes in all geochemical and sedimentological proxies. The amount of coarser sediment increases progressively to the top of the section. The 13 topmost layers represent alternations of silt/clay and sand. Both sulphur and organic carbon fluctuate exhibiting minima synchronous with increases in coarser sediment (Fig. 4, Fig. 9). The excursions are similarly present in the C/S-ratio with values often above 10 and even as high as 21.87 (Fig. 4, Fig. 9). Values of this magnitude would normally point towards a freshwater zone, but Berner and Raiswell (1984) pointed out that low levels of organic carbon make the C/S-ratio generally of little use in sediments with a high sand content.

*C. pelagicus* and *C. floridanus* decrease, while *R. minuta* and the content of other autochthonous taxa increase towards the top. The content of allochthonous taxa also increases. The lower content of *C. pelagicus* indicates reduced upwelling conditions. This decrease is further supported by an overall increase in nannoplankton diversity in this part of the section, as indicated by the Shannon–Wiener and dominance indices (Fig. 5). Higher influx of terrigenous material is indicated by high amounts of allochthonous taxa as well as an increase in small reticulofenestrids. Increases in warm water and ‘near shore’ taxa point towards a sea level fall.

At the top, increased freshwater influx, similar to Interval A, is indicated by an increase in near-shore taxa such as *R. minuta* (Haq, 1980) and also the raised amount of allochthonous taxa. Coastal proximity is reflected by a marked increase in accessory taxa such as *Sphenolithus*, *Helicosphaera* and *Coronosphaera*. Spikes of those taxa are predominantly recorded in the uppermost part of the section and can thus be correlated with the increased influx of coarser sediment (Fig. 5, Fig. 7, Fig. 9).

## 6 Conclusions

This study is the first attempt to use integrated proxy records (calcareous nannoplankton, geochemistry, sedimentology) to analyse high-resolution outcrop data of upper Burdigalian (Karpatian) sediments from the shelf of the Paratethys Sea. The sediments were biostratigraphically dated using calcareous nannofossils (NN4). A high-resolution section with a thickness of 940.5 mm was sampled continuously with a sampling distance of ~ 10 mm and analysed. The results document surprisingly rapid fluctuations of the palaeoenvironmental conditions within this Miocene shelf environment on a decadal to centennial scale. The study also shows that taphonomic processes and palaeoecological proxies are useful tools for the characterisation of small-scale, short-term fluctuations in the palaeoenvironment.

Cluster analysis and nMDS group the autochthonous nannofossils into four assemblages that reflect the ecological preferences of taxa. The results further indicate that, while certain taxa are likely contaminated by reworked specimens, a primary ecological signal is still preserved within the assemblages. Generally, the autochthonous assemblages are indicative of a cool to temperate (~ 14 to 17 °C) eutrophic near-shore environment with high terrigenous input and wind-driven upwelling, based on the presence of *C. pelagicus*, *R. haqii*, *R. minuta*, *H. carteri* and *C. floridanus* in all samples. Additionally, the low diversity of the assemblage suggests a dynamic near-shore setting with a strong variability in environmental conditions.

Subsequent Q-mode analyses (cluster analysis and nMDS) using both autochthonous and allochthonous assemblages revealed three distinct taphogroups with two sub-groups each. While similar in composition the taphogroups still reflect small-scale variations of the prevalent palaeoenvironmental conditions throughout the section. The distribution of these taphogroups is based on changes in relative sea level, fluctuations in upwelling intensity, primary productivity, water stratification and freshwater influx, which have influenced the composition of the nannofossil assemblages.

The distribution of the three taphogroups and respective sub-groups in combination with geochemical and sedimentological proxies allowed the definition of five intervals. The intervals reflect changes in the palaeoenvironment of a highly dynamic shallow marine setting that was subject to small-scale short-term climate variability on a centennial to decadal scale (Fig. 9):

The section begins with slightly freshwater-influenced marine sediments with strong terrigenous input. These initial conditions progress towards a more open marine setting reflected in geochemical proxies (sulphur, C/S-ratio) as well as the nannoplankton assemblages with an increase in *C. floridanus* and *C. pelagicus* (Fig. 7, Fig. 9.). While the geochemical proxies indicate more or less stable conditions for most of the section the nannoplankton assemblages indicate a shift towards cooler water temperatures, possibly due to an increase in upwelling conditions in the middle part of the section (Fig. 7, Fig. 9). Finally, the development in the uppermost part of the section points towards a decrease in water depth (Fig. 3). The nannoplankton taphocoenoses further support a fall in relative sea-level with elevated amounts of allochthonous taxa, as well as higher contents of near shore and stress taxa, such as *R. minuta* and *Helicosphaera* pointing towards a dynamic environment with high nutrient levels, most likely of terrestrial origin and high terrigenous input (Fig. 9).

## Appendix A Supplementary data

Taxonomic Appendix
