# Supplementary material for: High-resolution calcareous nannoplankton palaeoecology as a proxy for small-scale environmental changes in the Early Miocene
Source: Mar Micropaleontol. 2014 Sep;111:53–65. doi: 10.1016/j.marmicro.2014.06.005 (PMC4459667; doi:10.1016/j.marmicro.2014.06.005)
Supplement: Supplementary file 1 — Taxonomic Appendix [file mmc1.docx]

Supplement 1

Taxonomic appendix (after Perch-Nielsen, 1985a; 1985b; Bown, 1998)

Autochthonous taxa:

*Braarudosphaera bigelowii* (Gran and Braarud, 1935) emend. Deflandre, 1947a

*Calcidiscus leptoporus* (Murray and Blackman, 1898) Loeblich and Tappan, 1978

*Calcidiscus tropicus* Kamptner, 1956 sensu Gartner, 1992

*Coccolithus pelagicus* (Wallich, 1877) Schiller, 1930

*Coronocyclus nitescens* (Kamptner, 1963) Bramlette and Wilcoxon, 1967

*Coronosphaera mediterranea* (Lohmann, 1902) Gaarder in Gaarder and Heimdal (1977)

*Cyclicargolithus floridanus* Roth and Hay in Hay et al., 1967 Bukry, 1971a

*Discoaster deflandrei* Bramlette and Riedel, 1954

*Helicosphaera ampliaperta* Bramlette and Wilcoxon, 1967

*Helicosphaera carteri* (Wallich, 1877) Kamptner, 1954

*Helicosphaera euphratis* Haq, 1966

*Helicosphaera intermedia* Martini, 1965

*Helicosphaera mediterranea* Müller, 1981

*Helicosphaera obliqua* Bramlette and Wilcoxon, 1967

*Helicosphaera perch-nielseniae* (Haq, 1971) Jafar and Martini, 1975

*Helicosphaera scissura* Miller, 1981

*Micrantholithus vesper* Deflandre, 1950

*Pontosphaera discopora* (Schiller, 1925)

*Pontosphaera multipora* (Kamptner, 1948)

*Reticulofenestra haqii* Backman, 1978

*Reticulofenestra minuta* Roth, 1970

*Reticulofenestra pseudoumbilicus* (Gartner, 1967) Gartner, 1969c

*Sphenolithus heteromorphus* Deflandre 1953

*Sphenolithus moriformis* (Bronnimann and Stradner, 1960) Bramlette and Wilcoxon, 1967a

*Triquetrorhabdulus carinatus* Martini, 1965

*Umbilicosphaera jafari* Müller, 1974b

*Umbilicosphaera rotula* (Kamptner, 1956) Varol, 1982

Allochthonous taxa

Paleogene taxa

*Biscutum harrisonii* Varol, 1989b

*Chiasmolithus expansus* (Bramlette and Sullivan, 1961) Gartner, 1970

*Chiasmolithus solitus* (Bramlette and Sullivan, 1961) Locker, 1968

*Clausicoccus fenestratus* (Deflandre and Fert, 1954) Prins, 1979

*Clausicoccus vanheckiae* (Perch-Nielsen, 1986), De Kaenel and Villa, 1996

*Coccolithus formosus* (Kamptner, 1963) Wise, 1973

*Coccolithus subpertusus* (Hay and Mohler, 1967) van Heck and Prins, 1987

*Cribrocentrum* *reticulatum* (Gartner and Smith, 1967) Perch-Nielsen, 1971b

*Cruciplacolithus asymmetricus* van Heck and Prins, 1987

*Cruciplacolithus primus* Perch-Nielsen, 1977

*Cruciplacolithus tenuis* (Stradner, 1961) Hay and Mohler in Hay et al., 1967

*Cyclagelosphaera alta* Perch-Nielsen, 1979b

*Cyclicargolithus luminis* (Sullivan, 1965) Bukry, 1971a

*Discoaster araneus* Bukry, 1971

*Discoaster kuepperi* Stradner, 1959b

*Discoaster multiradiatus* Bramlette and Riedel, 1954

*Discoaster saipanensis* Bramlette and Riedel, 1954

*Helicosphaera bramlettei* (Müller, 1970)

*Helicosphaera lophota* (Bramlette and Sullivan, 1961)

*Helicosphaera seminulum* Bramlette and Sullivan, 1961

*Isthmolithus recurvus* Deflandre in Deflandre and Fert, 1954

*Lanternithus minutus* Stradner, 1962

*Micrantholithus attenuatus* Bramlette and Sullivan, 1961

*Neococcolithes protenus* (Bramlette and Sullivan, 1961) Black, 1967

*Pontosphaera enormis* (Locker, 1967) Perch-Nielsen, 1984

*Pontosphaera exilis* (Bramlette and Sullivan, 1961) Romein, 1979

*Pontosphaera rimosa* (Bramlette and Sullivan, 1961) Roth and Thierstein, 1972

*Praeprinsius* *dimorphosus* (Perch-Nielsen, 1969) Varol and Jakubowski, 1989

*Prinsius martinii* (Perch-Nielsen, 1969) Haq, 1971

*Reticulofenestra bisecta* (Hay, Mohler and Wade, 1966) Roth, 1970

*Reticulofenestra daviesii* (Haq, 1968) Haq, 1971

*Reticulofenestra dictyoda* (Deflandre in Deflandre and Fert, 1954) Stradner in Stradner and Edwards, 1968

*Reticulofenestra umbilicus* (Levin, 1965) Martini and Ritzkowski, 1968

*Sphenolithus capricornutus* Bukry and Percival, 1971

*Sphenolithus ciperoensis* Bramlette and Wilcoxon, 1967a

*Sphenolithus conicus* Bukry, 1971

*Sphenolithus radians* Delfandre in Grassé, 1952

*Sullivania* *danica* (Brotzen, 1959) Varol, 1992

*Toweius rotundus* Perch-Nielsen in Perch-Nielsen et al., 1978

*Transversopontis rectipons* (Haq, 1969) Roth, 1970

*Zygrhablithus bijugatus* (Deflandre in Deflandre and Fert, 1954) Deflandre, 1959

Cretaceous taxa

*Ahmuellerella octoradiata* (Górka, 1957) Reinhardt, 1966

*Arkhangelskiella confusa* Burnett, 1997b

*Arkhangelskiella cymbiformis* Vekshina, 1959

*Arkhangelskiella maastrichtiensis* Burnett, 1997b

*Biscutum coronum* Wind and Wise in Wise and Wind, 1977

*Biscutum melaniae* (Górka, 1957) Burnett, 1997

*Broinsonia parca* (Stradner, 1963) Bukry, 1969 ssp. *constricta* Hattner et al., 1980

*Broinsonia parca* (Stradner, 1963) Bukry, 1969 ssp. *exansa* Wise and Watkins in Wise, 1983

*Broinsonia parca* (Stradner, 1963) Bukry, 1969 ssp. *parca*

*Calculites obscurus* (Deflandre 1959) Prins and Sissingh in Sissingh 1977

*Calculites percenis* Jeremiah, 1996

*Cribrosphaerella ehrenbergii* (Arkhangelsky, 1912) Deflandre in Piveteau, 1952

*Cyclagelosphaera margerelii* Noël, 1965

*Cyclagelosphaera reinhardtii* (Perch-Nielsen, 1968) Romein, 1977

*Eiffellithus eximius* (Stover, 1966) Perch-Nielsen, 1968

*Eiffellithus gorkae* Reinhardt, 1965

*Eiffellithus turriseiffelii* (Deflandre in Deflandre and Fert, 1954) Reinhardt, 1965

*Gorkaea obliqueclausus* (Varol, 1991) Varol and Girgis, 1994

*Heteromarginatus bugensis*(Górka, 1957) Crux in Crux et al., 1982

*Lithastrinus grillii* Stradner, 1962.

*Lucianorhabdus cayeuxii* Deflandre, 1959

*Lucianorhabdus maleformis* Reinhardt, 1966

*Markalius* *inversus* (Deflandre in Deflandre and Fert, 1954) Bramlette and Martini, 1964

*Microrhabdulus belgicus* Hay and Towe, 1963

*Microrhabdulus undosus* Perch-Nielsen, 1973

*Micula decussata* Vekshina, 1959

*Micula murus* (Martini, 1961) Bukry, 1973c

*Micula prinsii* Perch-Nielsen, 1979a

*Micula staurophora* (Gardet, 1955) Stardner, 1963

*Owenia hillii* Crux, 1991b, see *I. compactus* Verbeek, 1976b

*Percivalia imperfossa* Black, 1971

*Placozygus fibuliformis* (Reinhardt, 1964) Hoffmann, 1970

*Placozygus sigmoides* (Bramlette and Sullivan, 1961) Bown and Young, 1997

*Podorhabdus? elkefensis* Perch-Nielsen, 1981c

*Prediscosphaera columnata* (Stover, 1966) Perch-Nielsen, 1984

*Prediscosphaera cretacea* (Arkhangelsky, 1912) Gartner, 1968

*Prediscosphaera microrhabdulina* Perch-Nielsen, 1973

*Prediscosphaera ponticula* (Bukry, 1969) Perch-Nielsen, 1984

*Psyktosphaera firthii* Pospichal and Wise, 1990

*Quadrum gartneri* Prins and Perch-Nielsen in Manivit et al., 1977

*Quadrum svabenickae* Burnett, 1997b

*Retecapsa crenulata* (Bramlette and Martini, 1964) Grün in Grün and Allemann, 1975

*Retecapsa ficula* (Stover, 1966) Burnett, 1997

*Rhagodiscus asper* (Stradner, 1963) Reinhardt, 1967

*Staurolithites flavus* Burnett, 1997b

*Staurolithites gausorhethium* (Hill, 1976) Varol and Girgis, 1994

*Staurolithites zoensis* Burnett, 1997b

*Tegumentum stradneri* Thierstein in Roth and Thierstein, 1972

*Tetrapodorhabdus decorus* (Deflandre in Deflandre and Fert, 1954) Wind and Wise in Wise and Wind, 1977

*Uniplanarius gothicus* (Deflandre, 1959) Hattner and Wise, 1980

*Uniplanarius sissinghii* Perch-Nielsen, 1986b

*Watznaueria barnesiae* (Black, 1959) Perch-Nielsen, 1968

*Watznaueria biporta* Bukry, 1969

*Watznaueria britannica* (Stradner, 1963) Reinhardt, 1964

*Watznaueria quadriradiata* Bukry, 1969

*Zeugrhabdotus bicrescenticus* (Stover, 1966) Burnett in Gale et al., 1996

*Zeugrhabdotus diplogrammus* (Deflandre in Deflandre and Fert, 1954) Burnett in Gale et al., 1996

*Zeugrhabdotus embergeri*(Noël, 1958) Perch-Nielsen, 1984

*Zeugrhabdotus scutula* (Bergen, 1994) Rutledge and Bown, 1996
